# Association between dietary knowledge and overweight/obesity in Chinese children and adolescents aged 8–18 years: a cross-sectional study

**DOI:** 10.1186/s12887-022-03618-2

**Published:** 2022-09-23

**Authors:** Lihong Wang, Jielian Zhuang, Heng Zhang, Weijuan Lu

**Affiliations:** 1 Department of Endocrinology and Metabolism, Children’s Hospital of Shanxi and Women Health Center of Shanxi, Taiyuan, 030000 Shanxi People’s Republic of China; 2 Department of Child Health Care, Wuxi Maternity and Child Health Care Hospital, No. 48 Huaishu lane, Liangxi District, Wuxi, 214002 Jiangsu People’s Republic of China

**Keywords:** Dietary knowledge, Overweight and obesity, Children

## Abstract

**Background:**

A lack of adequate dietary knowledge may result in poor health. The purpose of this study was to study the association between dietary knowledge and overweight/obesity in children and adolescents.

**Method:**

Data from the China Health and Nutrition Survey (CHNS) 2004, 2006, 2009, 2011, and 2015 were used in this cross-sectional study. The dietary knowledge of children and adolescents was evaluated by the questionnaire in the database. The overweight and obesity status was evaluated by body mass index (BMI). Cluster analysis was performed to establish different groups based on dietary knowledge level. Logistic regression analysis and subgroup analysis were conducted.

**Results:**

A total of 2,701 children and adolescents were finally selected. Cluster A (*n* = 837, 30.99%), Cluster B (*n* = 1,264, 46.80%) and Cluster C (*n* = 600, 22.21%) were high, medium and low dietary knowledge level, respectively. Participants with high dietary knowledge levels [OR = 0.56 (95%CI: 1.40–0.78)] may be negatively associated with overweight and obesity. Similar results were found among adolescents, males, females, people living in eastern and northeastern China, and rural areas, after adjusting for age, gender, geographic region, maternal education level, alcohol consumption, waist-to-hip ratio, systolic blood pressure and diastolic blood pressure.

**Conclusion:**

Improving the dietary knowledge level of children and adolescents was associated with decreased risk of overweight and obesity. Our study provided a theoretical basis for the relationship between dietary knowledge and overweight/obesity in Chinese children and adolescents and suggested strengthening the publicity and popularization of dietary knowledge in schools and communities.

**Supplementary Information:**

The online version contains supplementary material available at 10.1186/s12887-022-03618-2.

## Background

The incidence of overweight or obesity among children and adolescents worldwide is rising rapidly [1]. Being overweight or obese in children is related to multiple risk factors for advanced heart disease and other chronic diseases, including hyperlipidemia, hyperinsulinemia, hypertonicity, and early atherosclerosis [2, 3]. Diseases caused by obesity may impose a heavy burden on healthcare and cause serious social and economic consequences [4]. In view of the significant short-term and long-term health and social consequences, preventing childhood obesity is essential.

Family-based intervention projects have been carried out in children to prevent overweight or obesity in children or adolescents, with an emphasis on dietary knowledge [3, 5]. Dietary knowledge is one of the important factors to choose a healthy and nutritious diet [3]. Inappropriate dietary knowledge is one of the main causes of nutrition problems and has a negative impact on eating habits [6]. Personal dietary knowledge can affect his food choices, which may also affect his health [7]. The growth phase is a critical period to establish healthy behavior patterns to avoid obesogenic habits that may negatively affect future health. Parents’ dietary knowledge is a well-known factor affecting children’s diet, which is closely related to childhood and adolescent obesity, especially early obesity, as the current studies at home and abroad have shown [8, 9]. Previous studies explored the relationship between children's dietary knowledge and body mass index (BMI). Some studies showed significant associations between children’s dietary knowledge and BMI among Polish children and adolescents and American children [10, 11]. However, little is known about the association between children's dietary knowledge and overweight or obesity among Chinses children and adolescents. Dietary knowledge in children and adolescents is especially important given the issue of obesity in childhood and adolescents, and obesity in childhood predicts obesity in adulthood.

Therefore, the aim of this study was to identify different dietary knowledge levels and their association with overweight and obesity among Chinese children and adolescents. We also conducted subgroup analyses based on age, gender, and region to study the association between diet knowledge and overweight/obesity. The study from the Chinese population may serve as a reference for future research.

## Methods

### Study population

In this cross-sectional study, we used the data from the China Health and Nutrition Survey (CHNS) 2004, 2006, 2009, 2011, and 2015. The database collected information about the nutritional and health status of the Chinese population, as well as demographic and socio-economic data, and was established by the University of North Carolina at Chapel Hill and the Chinese Center for Disease Control and Prevention. The sampling method was a multi-stage randomized design in 15 provinces and megacities in 2004, 2006, 2009, 2011, and 2015 CHNS surveys.

**Fig. 1:**
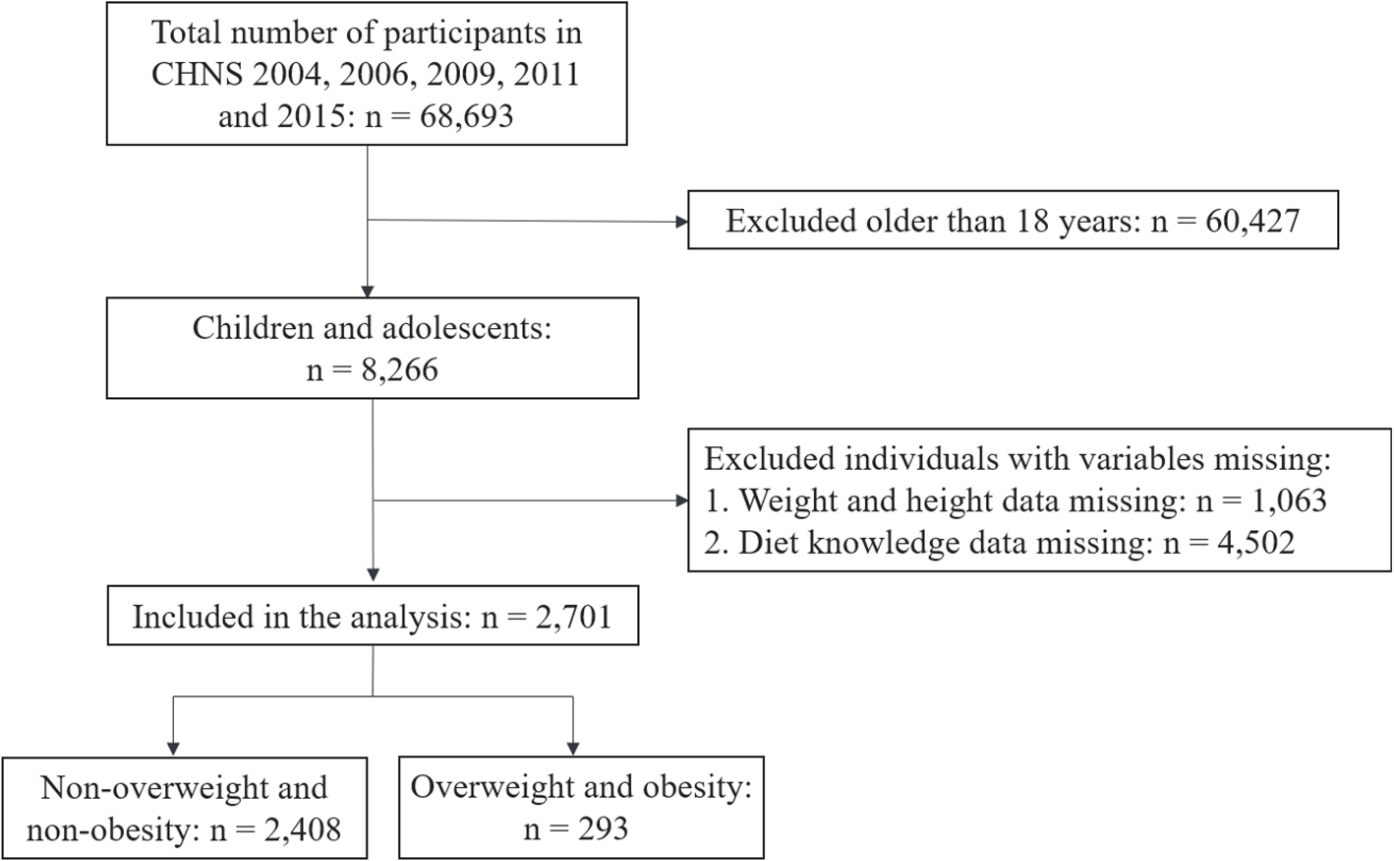
Flowchart of the systematic selection process

Children and adolescents aged 8–18 (*n* = 8266) were extracted from a total of 68,693 CHNS surveys in 2004, 2006, 2009, 2011 and 2015. After excluding those with missing height, weight characteristics (*n* = 1,063), and dietary knowledge information (*n* = 4,502), a total of 2,701 subjects were involved in the final analysis. The flow chart of the systematic selection process was shown in Fig. 1. This study used de-identified and publicly-available datasets from the official CHNS website (https://www.cpc.unc.edu/projects/china). Hence, approval from the Institutional Review Board of Wuxi Maternity and Child Health Care Hospital was not required.

### Overweight or obesity

Overweight or obesity was defined using age- and sex-specific BMI cut-off points, which was from the International Obesity TaskForce (IOTF) [12] (Supplementary Table 1). BMI was used as a surrogate indicator of obesity because it was widely used in clinical and epidemiological classification of children's weight status [13].

### Dietary knowledge

Dietary knowledge consists of 17 questions to which the responses were strongly disagree, somewhat disagree, neutral, somewhat agree, or strongly agree. The questions are not asked about the participants’ actual habits, but about their perception of dietary knowledge. The description of Q1-Q17 and its code are shown in Table 1.

**Table 1:** Dietary knowledge questions in the CHNS questionnaire

No	Questions	Code
Q1	Choosing a diet with a lot of fresh fruits and vegetables is good for one’s health	LOF
Q2	Eating a lot of sugar is good for one’s health	LOS
Q3	Eating a variety of foods is good for one’s health	VOF
Q4	Choosing a diet high in fat is good for one’s health	DHIF
Q5	Choosing a diet with a lot of staple foods [rice and rice products and wheat and wheat products] is not good for one’s health	LOSF
Q6	Consuming a lot of animal products daily (fish, poultry, eggs and lean meat) is good for one’s health	LOAP
Q7	Reducing the amount of fatty meat and animal fat in the diet is good for one’s health	AAM
Q8	Consuming milk and dairy products is good for one’s health	MDP
Q9	Consuming beans and bean products is good for one’s health	BBP
Q10	Physical activities are good for one’s health	PA
Q11	Sweaty sports or other intense physical activities are not good for one’s health	IPA
Q12	The heavier one’s body is, the healthier he or she is	HB
Q13	Eating salty foods can cause hypertension	ESF
Q14	Refined grains (rice and wheat flour) contain more vitamins and materials than unrefined grains	RG
Q15	Lard is healthier than vegetable oils	LA
Q16	Vegetables contain more starch than staple foods (rice or wheat flour)	VE
Q17	Eggs and milk are the important sources of high-quality protein	EAM

### Potential covariates

Potential covariates including age, gender, geographic region, type of household registration, maternal education level, smoking, alcohol consumption, waist-to-hip ratio, systolic blood pressure (SBP), and diastolic blood pressure (DBP) were collected. Geographic regions were divided into four regions, eastern China, central China, western China and northeastern China. Eastern China includes Beijing and Shanghai, Jiangsu, Shandong, and Zhejiang provinces; central China includes the Henan, Hubei and Hunan provinces; western China includes Guizhou, Guangxi, Shanxi, and Yunnan provinces, and Chongqing; northeastern China includes Liaoning and Heilongjiang provinces. Urban and rural are the two types of household registration. Maternal education level was classified as below primary school, primary school, middle school, high school, and above. Variables with significant differences (*P* < 0.05) in univariate analysis were included as covariates in multivariate analysis.

### Statistical analysis

Normally distributed measurement data were described by mean ± standard deviation (SD), and a t-test was used to compare between groups. Counting data were described in terms of cases and the constituent ratio [n (%)], and the chi-square test or Fisher’s exact probability method was used for comparison between groups. Multiple imputations (R: mice) were performed on missing data, and sensitivity analysis was performed on the differences between the data before and after imputation. In order to achieve the purpose of the study, firstly, the basic characteristics of the study population were compared, and the possible confounders related to overweight or obesity in children and adolescents were explored (*P* < 0.05). Secondly, K-means cluster analysis was performed on the dietary knowledge of the study population [14]. The number of clusters identified was specified in the first step and random initial seed and ten iterations were used to further refine the preliminary solution by optimizing the classification. The characteristics of the participants in groups with different levels of dietary knowledge were shown. Thirdly, the selected confounders (age, gender, geographic region, maternal education level, alcohol consumption, waist-to-hip ratio, SBP, and DBP) were included as covariates and the multivariate logistic regression analysis was used to explore the association between dietary knowledge level and overweight and obesity. Finally, a subgroup analysis was conducted based on age [children (8–12 years old), and adolescents (13–18 years old)], gender (males and females), geographic region (eastern China, western China, central China, and northeastern China) and residential areas (urban and rural). The age division of children and adolescents in China is mainly based on primary and secondary schools, which are divided into five grades (grades 1–6 with students aged 6–12) and three grades (grades 7–9 with students aged 13–16), respectively. In our study population, children were classified as 8–12 years old, and adolescents as 13–18 years old.

All statistical tests used two-sided tests, *P* < 0.05 was a statistically significant difference in the test. The statistical analysis was completed using SAS v. 9.4 (SAS Institute, Cary, North Carolina) and R v. 4.20 (R Foundation for Statistical Computing, Vienna, Austria).

## Results

### Characteristics of the study population

Socio-demographic information was presented in Table 2. Among 2,701 participants, 1,425 (52.76%) were males and 1,276 (47.24%) were females. The mean age was 14.63 ± 1.96 years. The proportion of participants in eastern China, central China, western China, and northeastern China was 631 (23.36%), 712 (26.36%), 795 (29.43%) and 563 (20.84%), respectively. Most participants were in the rural area (62.87%). Among the maternal education level, 1,077 (39.87%) participants were middle school graduates, and 72 (26.77%) were high school graduates or above graduates. There were 293 (10.85%) participants who were overweight or obese. Comparing the characteristics of the population before and after data imputation, there were no significant differences were observed (*P* > 0.05) (Supplementary Table 2).

**Table 2:** Descriptive characteristics of children and adolescents by body mass index (BMI) status

Characteristic	Total (*n* = 2,701)	Groups		Statistic	*P*
		Non-overweight or non-obesity (*n* = 2,408)	Overweight or obesity (*n* = 293)		
Age, years, Mean ± SD	14.63 ± 1.96	14.68 ± 1.95	14.24 ± 1.94	t = 3.64	< 0.001
Gender, n (%)				χ^2^ = 11.547	< 0.001
Male	1425 (52.76)	1243 (51.62)	182 (62.12)		
Female	1276 (47.24)	1165 (48.38)	111 (37.88)		
Geographic region^a^, n (%)				χ^2^ = 54.050	< 0.001
Eastern China	631 (23.36)	520 (21.59)	111 (37.88)		
Central China	712 (26.36)	648 (26.91)	64 (21.84)		
Western China	795 (29.43)	748 (31.06)	47 (16.04)		
Northeastern China	563 (20.84)	492 (20.43)	71 (24.23)		
Residential areas, n (%)				χ^2^ = 0.630	0.428
Urban	1003 (37.13)	888 (36.88)	115 (39.25)		
Rural	1698 (62.87)	1520 (63.12)	178 (60.75)		
Maternal education level, n (%)				Z = 4.189	< 0.001
Below primary school	356 (13.18)	327 (13.58)	29 (9.90)		
Primary school	545 (20.18)	499 (20.72)	46 (15.70)		
Middle school	1077 (39.87)	968 (40.20)	109 (37.20)		
High school and above	723 (26.77)	614 (25.50)	109 (37.20)		
Smoking, n (%)	95 (3.52)	81 (3.36)	14 (4.78)	χ^2^ = 1.540	0.215
Alcohol consumption, n (%)	390 (14.44)	329 (13.66)	61 (20.82)	χ^2^ = 10.828	< 0.001
Waist-to-hip ratio, Mean ± SD	0.82 ± 0.09	0.82 ± 0.09	0.87 ± 0.08	t = -10.96	< 0.001
SBP, mmHg, Mean ± SD	106.05 ± 11.49	105.41 ± 11.27	111.29 ± 11.89	t = -8.38	< 0.001
DBP, mmHg, Mean ± SD	69.53 ± 8.52	69.20 ± 8.50	72.25 ± 8.25	t = -5.82	< 0.001

*SD* Standard deviation, *SBP* Systolic blood pressure, *DBP* Diastolic blood pressure

^a^Eastern China includes: the city of Beijing and Shanghai, the province of Jiangsu, Shandong and Zhejiang; Central China includes: the province of Henan, Hubei and Hunan; Western China includes: the province of Guizhou, Guangxi, Shanxi and Yunnan, the city of Chongqing; Northeastern China includes: the province of Liaoning and Heilongjiang

### Comparison of non-overweight/non-obesity group and overweight/obesity group

Compared with participants in the non-overweight or non-obesity group, participants in the overweight or obesity group were more likely to be male (62.12% vs. 51.62%, *P* < 0.001), living in eastern China (37.88% vs. 21.59%, *P* < 0.001) or northeastern China (24.23% vs. 20.43%, *P* < 0.001), to have more alcohol consumption (20.82% vs. 13.66%, *P* < 0.001) and to have mothers with high school or above education level (37.20% vs. 25.50%, *P* < 0.001), and showed significantly greater values for waist-to-hip ratio (0.87 ± 0.08 vs. 0.82 ± 0.09, *P* < 0.001), SBP (111.29 ± 11.89 vs. 105.41 ± 11.27 mmHg, *P* < 0.001) and DBP (72.25 ± 8.25 vs. 69.20 ± 8.50 mmHg, *P* < 0.001). There was a significant difference in age [14.24 ± 1.94 years old in the overweight or obesity group vs. 14.68 ± 1.95 years old in the non-overweight or non-obesity group (*P* < 0.001)]. No differences were found in type of household registration and smoking. The comparison is shown in Table 2. These results showed that age, gender, geographic region, maternal education level, alcohol consumption, waist-to-hip ratio, SBP, and DBP were covariates.

### Clustering of diet knowledge level

**Fig. 2:**
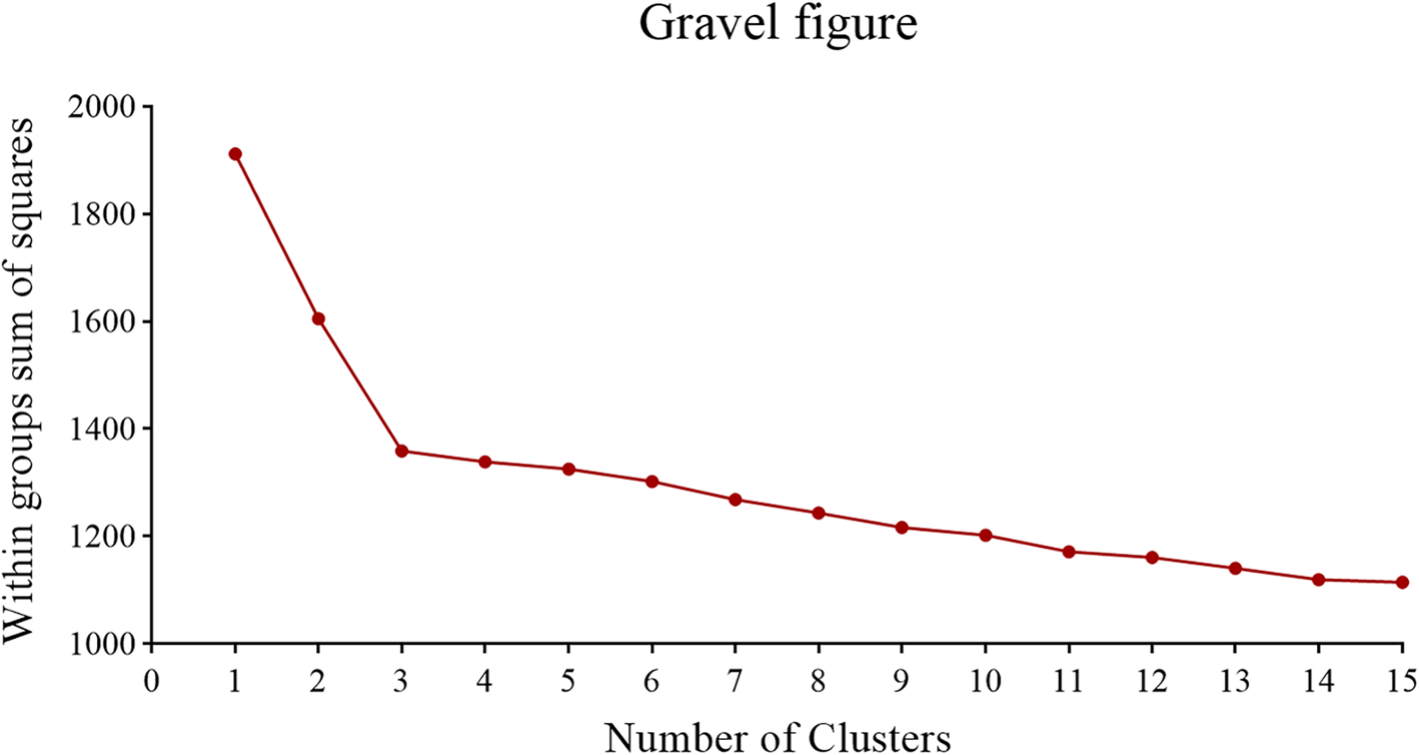
Gravel diagram of the K-means cluster analysis

**Fig. 3:**
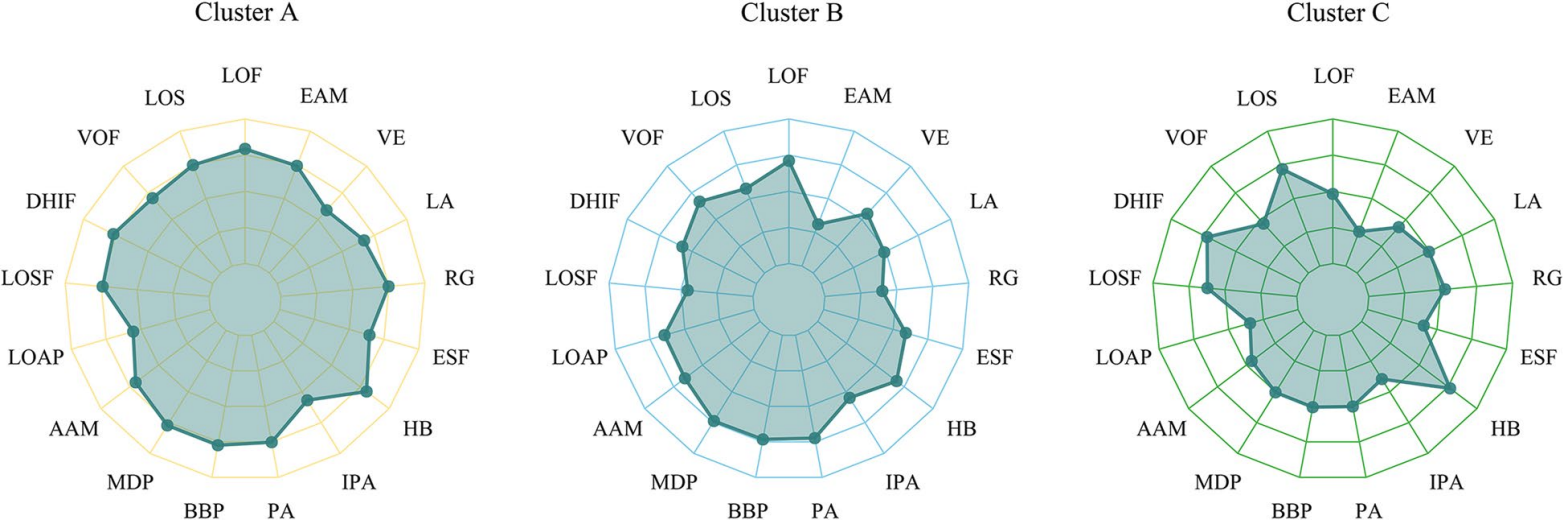
Cluster characteristics based on the measurement of diet knowledge (cluster A, cluster B, cluster C). LOF, LOS, VOF, DHIF, LOSF, LOAP, AAM, MDP, BBP, PA, IPA, HB, ESF, RG, LA, VE, and EAM correspond to the code of Q1 to Q17 in Table 1, respectively

According to the gravel diagram of the K-means cluster analysis, the three clusters solution for dietary knowledge was considered the most interpretable and stable classification (Fig. 2). The answer scores of the 17 questions are shown in Table 1, the mean scores of Cluster A, Cluster B, and Cluster C were calculated respectively. The mean total scores of the three Clusters A, B, and C were 3.84, 3.36, and 3.02, respectively. Cluster A, Cluster B, and Cluster C were characterized by high, medium, and low dietary knowledge levels, respectively. Figure 3 shows the specific characteristics of each cluster.

Cluster A included 837 (30.99%) children and adolescents, Cluster C included 600 (22.21%) children and adolescents, and Cluster B comprised 1,264 (46.80%) children and adolescents between Cluster A and Cluster B. Characteristics of Cluster A’s, Cluster B’s, and Cluster C’s participants are shown in Table 3. The mean ± SD ages of the participants in Cluster A, Cluster B, and Cluster C were 14.74 ± 1.97, 14.66 ± 1.94, and 14.44 ± 1.95, respectively (*P* = 0.012). There were 438 (52.33%) males and 399 (47.67%) females in Cluster A, 641 (50.71%) males and 623 (49.29%) females in Cluster B, and 346 (57.67%) males and 254 (42.33%) females in Cluster C (*P* = 0.018).

**Table 3:** Characteristics of Cluster A’s, Cluster B’s and Cluster C’s participants

Characteristic	Total (*n* = 2,701)	Cluster A (*n* = 837)	Cluster B (*n* = 1264)	Cluster C (*n* = 600)	Statistic	*P*
Age, years, Mean ± SD	14.63 ± 1.96	14.74 ± 1.97	14.66 ± 1.94	14.44 ± 1.95	F = 4.412	0.012
Gender, n (%)					χ^2^ = 7.985	0.018
Male	1425 (52.76)	438 (52.33)	641 (50.71)	346 (57.67)		
Female	1276 (47.24)	399 (47.67)	623 (49.29)	254 (42.33)		
Geographic region^a^, n (%)					χ^2^ = 110.382	< 0.001
Eastern China	631 (23.36)	148 (17.68)	364 (28.80)	119 (19.83)		
Central China	712 (26.36)	273 (32.62)	254 (20.09)	185 (30.83)		
Western China	795 (29.43)	194 (23.18)	391 (30.93)	210 (35.00)		
Northeastern China	563 (20.84)	222 (26.52)	255 (20.17)	86 (14.33)		
Residential areas, n (%)					χ^2^ = 2.422	0.298
Urban	1003 (37.13)	293 (35.01)	484 (38.29)	226 (37.67)		
Rural	1698 (62.87)	544 (64.99)	780 (61.71)	374 (62.33)		
Maternal education level, n (%)					χ^2^ = 54.051	< 0.001
Below primary school	356 (13.18)	126 (15.05)	132 (10.44)	98 (16.33)		
Primary school	545 (20.18)	185 (22.10)	225 (17.80)	135 (22.50)		
Middle school	1077 (39.87)	353 (42.17)	487 (38.53)	237 (39.50)		
High school and above	723 (26.77)	173 (20.67)	420 (33.23)	130 (21.67)		
Smoking, n (%)	95 (3.52)	32 (3.82)	40 (3.16)	23 (3.83)	χ^2^ = 0.871	0.647
Alcohol consumption, n (%)	390 (14.44)	74 (8.84)	196 (15.51)	120 (20.00)	χ^2^ = 37.415	< 0.001
Waist-to-hip ratio, Mean ± SD	0.82 ± 0.09	0.82 ± 0.08	0.82 ± 0.10	0.83 ± 0.09	F = 1.337	0.263
SBP, mmHg, Mean ± SD	106.05 ± 11.49	106.55 ± 11.26	105.88 ± 11.20	105.73 ± 12.34	F = 1.158	0.314
DBP, mmHg, Mean ± SD	69.53 ± 8.52	69.79 ± 8.58	69.51 ± 8.21	69.23 ± 9.06	F = 0.776	0.460

Cluster A: high diet knowledge level; Cluster B: medium diet knowledge level; Cluster C: low diet knowledge level

*SD* Standard deviation, *SBP* Systolic blood pressure, *DBP* Diastolic blood pressure

^a^Eastern China includes: the city of Beijing and Shanghai, the province of Jiangsu, Shandong and Zhejiang; Central China includes: the province of Henan, Hubei and Hunan; Western China includes: the province of Guizhou, Guangxi, Shanxi and Yunnan, the city of Chongqing; Northeastern China includes: the province of Liaoning and Heilongjiang

### The association between diet knowledge and overweight/obesity

In Table 4, compared with Cluster B (medium dietary knowledge level), Cluster A (high dietary knowledge level) [odds ratio (OR) with 95% confidence interval (CI) of 0.56, 1.40–0.78)] was negatively associated with overweight and obesity after adjusting for age, gender, geographic region, maternal education level, alcohol consumption, waist-to-hip ratio, SBP, and DBP (Table 4). No significant association was found between Cluster C (low dietary knowledge level) and overweight and obesity, compared with Cluster B. When the study population was divided into non-overweight or non-obesity, overweight and obesity groups, the multivariate model showed that the results were similar to normal weight, overweight, or obese two groups (Supplementary Table 3).

**Table 4:** The association between diet knowledge and overweight/obesity in children and adolescents

	Model 1		Model 2		Model 3	
	OR (95%CI)	*P*	OR (95%CI)	*P*	OR (95%CI)	*P*
Diet knowledge						
Cluster B	Ref		Ref		Ref	
Cluster A	0.54 (0.39–0.74)	< 0.001	0.54 (0.39–0.74)	< 0.001	0.56 (0.40–0.78)	< 0.001
Cluster C	1.29 (0.97–1.71)	0.082	1.22 (0.92–1.63)	0.170	1.31 (0.97–1.79)	0.083

Model 1: Univariate logistic regression analysis;

Model 2: Adjustment for age and gender;

Model 3: Adjustment for age, gender, geographic region, maternal education level, alcohol consumption, waist-to-hip ratio, systolic blood pressure and diastolic blood pressure

Cluster A: high diet knowledge level; Cluster B: medium diet knowledge level; Cluster C: low diet knowledge level

*Ref* Reference, *OR* Odds ratio, *CI* Confidence interval

Using Cluster B as the reference, Cluster A was found to be negatively associated with overweight and obesity among adolescents (OR = 0.54, 95%CI: 0.36–0.80), males (OR = 0.58, 95%CI: 0.37–0.90), females (OR = 0.50, 95%CI: 0.29–0.88), living eastern China (OR = 0.51, 95%CI: 0.26–0.99), living northeastern China (OR = 0.37, 95%CI: 0.19–0.71), and living rural area (OR = 0.38, 95%CI: 0.24–0.60) in Model 3 (Table 5).

**Table 5:** The association between diet knowledge and overweight/obesity in different age, gender, geographic region, and residential areas

Diet knowledge	Cases (n)	Model 1		Model 2		Model 3	
		OR (95%CI)	*P*	OR (95%CI)	*P*	OR (95%CI)	*P*
Children (8–12 years old)							
Cluster B	221	Ref		Ref		Ref	
Cluster A	125	0.64 (0.34–1.21)	0.167	0.67 (0.35–1.29)	0.233	0.71 (0.35–1.46)	0.357
Cluster C	120	0.93 (0.52–1.69)	0.819	0.92 (0.51–1.67)	0.791	1.10 (0.58–2.08)	0.769
Adolescents (13–18 years old)							
Cluster B	1,043	Ref		Ref		Ref	
Cluster A	712	0.52 (0.36–0.75)	< 0.001	0.51 (0.35–0.74)	< 0.001	0.54 (0.36–0.80)	0.002
Cluster C	480	1.40 (1.01–1.94)	0.042	1.33 (0.96–1.85)	0.084	1.43 (1.00–2.05)	0.048
Male							
Cluster B	641	Ref		Ref		Ref	
Cluster A	438	0.53 (0.35–0.80)	0.002	0.54 (0.36–0.82)	0.004	0.58 (0.37–0.90)	0.015
Cluster C	346	1.21 (0.84–1.73)	0.306	1.19 (0.83–1.71)	0.353	1.31 (0.88–1.94)	0.178
Female							
Cluster B	623	Ref		Ref		Ref	
Cluster A	399	0.53 (0.32–0.89)	0.016	0.52 (0.31–0.87)	0.013	0.50 (0.29–0.88)	0.016
Cluster C	254	1.33 (0.84–2.11)	0.227	1.28 (0.80–2.04)	0.298	1.31 (0.79–2.15)	0.297
Eastern China^1^							
Cluster B	364	Ref		Ref		Ref	
Cluster A	148	0.52 (0.29–0.94)	0.031	0.51 (0.28–0.92)	0.027	0.51 (0.26–0.99)	0.047
Cluster C	119	1.62 (0.99–2.64)	0.053	1.54 (0.94–2.52)	0.088	1.20 (0.70–2.04)	0.512
Central China^2^							
Cluster B	254	Ref		Ref		Ref	
Cluster A	273	0.83 (0.44–1.58)	0.572	0.82 (0.43–1.57)	0.553	0.91 (0.46–1.79)	0.774
Cluster C	185	1.65 (0.89–3.07)	0.111	1.49 (0.80–2.79)	0.212	1.68 (0.87–3.24)	0.125
Western China^3^							
Cluster B	391	Ref		Ref		Ref	
Cluster A	194	0.39 (0.15–1.03)	0.057	0.37 (0.14–1.00)	0.049	0.39 (0.14–1.06)	0.064
Cluster C	210	1.29 (0.68–2.45)	0.436	1.17 (0.62–2.24)	0.626	1.34 (0.68–2.65)	0.396
Northeastern China^4^							
Cluster B	255	Ref		Ref		Ref	
Cluster A	222	0.47 (0.26–0.87)	0.015	0.48 (0.26–0.88)	0.018	0.37 (0.19–0.71)	0.003
Cluster C	86	1.31 (0.69–2.48)	0.417	1.45 (0.75–2.78)	0.270	1.16 (0.57–2.38)	0.689
Urban							
Cluster B	484	Ref					
Cluster A	293	0.71 (0.43–1.17)	0.181	0.68 (0.41–1.12)	0.132	0.91 (0.53–1.55)	0.733
Cluster C	226	1.35 (0.86–2.14)	0.196	1.22 (0.77–1.95)	0.400	1.45 (0.88–2.39)	0.142
Rural							
Cluster B	780	Ref					
Cluster A	544	0.45 (0.29–0.68)	< 0.001	0.45 (0.30–0.69)	< 0.001	0.38 (0.24–0.60)	< 0.001
Cluster C	374	1.25 (0.87–1.79)	0.231	1.21 (0.85–1.74)	0.295	1.28 (0.86–1.91)	0.229

Cluster A: high diet knowledge level; Cluster B: medium diet knowledge level; Cluster C: low diet knowledge level

Model 1: Univariate logistic regression analysis

Model 2: Adjustment for age and gender

Model 3: Adjustment for age, gender, geographic region, maternal education level, alcohol consumption, waist-to-hip ratio, systolic blood pressure and diastolic blood pressure

*Ref* Reference

^1^Eastern China includes: the city of Beijing and Shanghai, the province of Jiangsu, Shandong and Zhejiang

^2^Central China includes: the province of Henan, Hubei and Hunan

^3^Western China includes: the province of Guizhou, Guangxi, Shanxi and Yunnan, the city of Chongqing

^4^Northeastern China includes: the province of Liaoning and Heilongjiang

## Discussion

This study showed that dietary knowledge in the studied children and adolescents was clustered into three differential groups including Cluster A (high diet knowledge level), Cluster B (medium diet knowledge level), and Cluster C (low diet knowledge level). Compared with Cluster B, Cluster A was negatively associated with overweight and obesity, similar results were also found among adolescents, males, females, and the participants living in eastern China, northeastern China, and rural areas.

In our study, almost 80% of the children and adolescents had a broad concept of healthy dietary [high diet knowledge level (30.99%) and medium dietary knowledge level (46.80%)]. However, low dietary knowledge still accounted for 22.21% of the study population. Even the children and adolescents in Cluster A performed well on most questions, but not as well on Q6 [Consuming a lot of animal products daily (fish, poultry, eggs, and lean meat) is good for one’s health], Q11 (Sweaty sports or other intense physical activities are not good for one’s health) and Q16 [Vegetables contain more starch than staple foods (rice or wheat flour)]. China’s economy is developing so rapid that various animal products have become commonplace. Children’s perception of food in the family environment may have led to a bias in Q6 [15]. The results suggested that children and adolescents should take appropriate amounts of animal products although they are at a critical period of growth and development [16]. The acceleration of urbanization, the increasing burden of schoolwork and the lack of outdoor sports for children and adolescents may lead to misunderstanding of Q11 [17–19]. For Q16, it may be because children and adolescents have not received systematic and comprehensive nutrition and health education. Therefore, it is necessary to provide children and adolescents with essential knowledge of a healthy diet [20]. The knowledge could be spread via families, schools, or communities by integrating dietary knowledge with other core subjects, such as language. In addition, although the children and adolescents in Cluster C were not as good as those in Cluster A and B in dietary knowledge, they performed better in Q4 (Choosing a high-fat diet is good for one’s health) and Q12 (The heavier one’s body is, the healthier he or she is). This may be due to the attention and intervention on childhood obesity, which has a subtle impact on children and adolescents [19, 21].

The childhood and adolescence period are critical periods for growth, as well as learning knowledge and developing good habits [21]. Promoting and learning about dietary knowledge are particularly important for the health of children and adolescents [3]. Failure to understand dietary knowledge may have a negative impact on dietary behavior and healthy growth. Previous studies found that there was a correlation between the level of dietary knowledge of children or adolescents and overweight/obesity [10, 11, 22]. A significant correlation between the BMI of adolescents and their dietary knowledge score was found in Lebanese adolescents aged 15–18 years [22]. In a Polish study consisting of 1,515 children and adolescents aged 6–18 years, the authors showed that a high percentage of obese/overweight children was associated with insufficient knowledge of diet, which may consequently increase the risk of cardiovascular diseases in the adult population [10]. Similar results were found in the US. preschoolers [11]. Similarly, Sun et al. reported the impact of dietary knowledge on obesity in Chinses adults from CHNS [23]. As for children’s dietary knowledge and their intake, there are few studies on the association between dietary knowledge and overweight or obesity in Chinese children or adolescents. Herein, our results showed that after adjusting age, gender, geographic region, maternal education level, alcohol consumption, waist-to-hip ratio, SBP, and DBP, the risk of overweight and obesity in children and adolescents with high dietary knowledge levels was 0.56 times lower than that of those with medium dietary knowledge levels. Although no significant differences were found between low dietary knowledge and overweight and obesity compared with medium dietary knowledge, this also suggested that improving dietary knowledge was critical for children and adolescents. These results provided a theoretical basis in the population of Chinese children and adolescents. Children and adolescents with a high level of dietary knowledge are well aware of the importance of healthy eating, so they may guide their eating styles, take the initiative to form healthy behaviors and reduce the occurrence of overweight and obesity [4].

In the subgroup analysis, we found that different dietary knowledge levels were not associated with overweight and obesity in children aged 8–12, but were significantly associated with adolescents. It may be because children have a low level of obesity-related knowledge are susceptible to food temptations and have poor self-control ability, while adolescents have a richer health knowledge system and strong self-discipline [24]. Children and adolescents in eastern and northeastern China with high dietary knowledge were associated with decreased risks of overweight and obesity compared with those with medium dietary knowledge levels, while the association between dietary knowledge and overweight/obesity was not been observed among those in central China. Due to the imbalance of socio-economic development, socio-economic changes may be heterogeneous in different geographic regions [25]. Eastern and northeastern China include the capital Beijing, Shanghai, Jiangsu Province, Liaoning provinces, and others whose economic development levels are at the forefront of China. Studies have shown that the influence of socioeconomic status on obesity has significant geographic significance [25], suggesting that we should focus on overweight and obesity among children and adolescents in eastern and northeastern China. Moreover, high dietary knowledge levels decreased the risk of overweight and obesity compared with medium dietary knowledge levels in rural areas. Children and adolescents in rural areas may lack better dietary knowledge resources, have a low possibility to acquire dietary knowledge from the media, and have fewer opportunities to visit nutritionists [26], suggesting dietary knowledge, guidance and intervention needs to be provided.

To improve the dietary knowledge of children and adolescents better and reduce the incidence of overweight and obesity from the perspective of diet, the following interventions could be taken. Schools could set up dietary health education courses and carry out health knowledge lectures, so that children and adolescents have more opportunities to learn systemic and rich dietary knowledge [27] and develop healthy diet patterns, which can benefit children and adolescents for life and affect the eating habits of the next generation [3, 28]. In addition, dietary knowledge and dietary behaviors of children and adolescents should be monitored regularly, and timely interventions should be implemented [19]. Parents should jointly supervise children and adolescents to learn dietary knowledge, raise awareness and promote behaviors changes in a healthy and beneficial direction [20, 29].

The strengths of this study were as follows. Firstly, the study performed a cluster analysis on the dietary knowledge level of children and adolescents analyzed its relationship with overweight and obesity and provided a theoretical basis for the healthy growth of Chinese children and adolescents. Secondly, the questions of dietary knowledge used in our study were derived from the Chinese Dietary Nutrition Guidelines, which were representative and credible. However, there were a few limitations in our study. First, we used cross-sectional data rather than longitudinal data. There may be temporal influencing factors on overweight and obesity over time. Prospective longitudinal studies are needed to investigate the risk factors that may contribute to the development of overweight and obesity among Chinese children and adolescents. Second, relevant literature showed that overweight and obesity may also be related to genetic factors [29], but this study did not examine them due to data constraints.

## Conclusion

In the present study, children and adolescents with high dietary knowledge levels were negatively associated with overweight and obesity compared with those with medium dietary knowledge levels, similar results were also found among adolescents, males, females, eastern, northeastern, and rural populations. The results of the study may provide evidence-based support for preventive health care to promote the normal growth and development of children and adolescents, and reduce the risk of diet-related overweight and obesity through early and appropriate improvement of dietary knowledge.

## Supplementary Information

**Additional file 1:** **Supplementary Table 1.** International cut off points for body mass index for overweight and obesity by sex between 2 and 18 years, defined to pass through body mass index of 25 and 30 kg/m2 at age 18, obtained by averaging data from Brazil, Great Britain, Hong Kong, Netherlands, Singapore, and United States [1]. **Supplementary Table 2.** Sensitivity analysis of interpolation data. **Supplementary Table 3.** The association between diet knowledge and overweight and obesity in children and adolescents.

## Data Availability

The datasets generated and/or analyzed during the current study are available in the CHNS database, https://www.cpc.unc.edu/projects/china.

## Abbreviations

BMI: Body mass index
CHNS: China Health and Nutrition Survey
IOTF: International Obesity TaskForce
SBP: Systolic blood pressure
DBP: Diastolic blood pressure
Mean ± SD: Mean ± standard deviation
n (%): Constituent ratio
