# Supplementary material for: Association between dietary knowledge and overweight/obesity in Chinese children and adolescents aged 8–18 years: a cross-sectional study
Source: BMC Pediatr. 2022 Sep 23;22:558. doi: 10.1186/s12887-022-03618-2 (PMC9502888; doi:10.1186/s12887-022-03618-2)
Supplement: Supplementary file 1 — Additional file 1: Supplementary Table 1. International cut off points for body mass index for overweight and obesity by sex between 2 and 18 years, defined to pass through body mass index of 25 and 30 kg/m2 at age 18, obtained by averaging data from Brazil, Great Britain, Hong Kong, Netherlands, Singapore, and United States [1]. Supplementary Table 2. Sensitivity analysis of interpolation data. Supplementary Table 3. The association between diet knowledge and overweight and obesity in children and adolescents. [file 12887_2022_3618_MOESM1_ESM.docx]

Supplementary Table 1 International cut off points for body mass index for overweight and obesity by sex between 2 and 18 years, defined to pass through body mass index of 25 and 30 kg/m2 at age 18, obtained by averaging data from Brazil, Great Britain, Hong Kong, Netherlands, Singapore, and United States [1]

| Age, years | BMI 25 kg/m^2^ | | BMI 30 kg/m^2^ | |
| --- | --- | --- | --- | --- |
|  | Males | Females | Males | Females |
| 2 | 18.41 | 18.02 | 20.09 | 19.81 |
| 2.5 | 18.13 | 17.76 | 19.80 | 19.55 |
| 3 | 17.89 | 17.56 | 19.57 | 19.36 |
| 3.5 | 17.69 | 17.40 | 19.39 | 19.23 |
| 4 | 17.55 | 17.28 | 19.29 | 19.15 |
| 4.5 | 17.47 | 17.19 | 19.26 | 19.12 |
| 5 | 17.42 | 17.15 | 19.30 | 19.17 |
| 5.5 | 17.45 | 17.20 | 19.47 | 19.34 |
| 6 | 17.55 | 17.34 | 19.78 | 19.65 |
| 6.5 | 17.71 | 17.53 | 20.23 | 20.08 |
| 7 | 17.92 | 17.75 | 20.63 | 20.51 |
| 7.5 | 18.16 | 18.03 | 21.09 | 21.01 |
| 8 | 18.44 | 18.35 | 21.60 | 21.57 |
| 8.5 | 18.76 | 18.69 | 22.17 | 22.18 |
| 9 | 19.10 | 19.07 | 22.77 | 22.81 |
| 9.5 | 19.46 | 19.45 | 23.39 | 23.46 |
| 10 | 19.84 | 19.86 | 24.00 | 24.11 |
| 10.5 | 20.20 | 20.29 | 24.57 | 24.77 |
| 11 | 20.55 | 20.74 | 25.10 | 25.42 |
| 11.5 | 20.89 | 21.20 | 25.58 | 26.05 |
| 12 | 21.22 | 21.68 | 26.02 | 26.67 |
| 12.5 | 21.56 | 22.14 | 26.43 | 27.24 |
| 13 | 21.91 | 22.58 | 26.84 | 27.76 |
| 13.5 | 22.27 | 22.98 | 27.25 | 28.20 |
| 14 | 22.62 | 23.34 | 27.63 | 28.57 |
| 14.5 | 22.96 | 23.66 | 27.98 | 28.87 |
| 15 | 23.29 | 23.94 | 28.30 | 29.11 |
| 15.5 | 23.60 | 24.17 | 28.60 | 29.29 |
| 16 | 23.90 | 24.37 | 28.88 | 29.43 |
| 16.5 | 24.19 | 24.54 | 29.14 | 29.56 |
| 17 | 24.46 | 24.70 | 29.41 | 29.69 |
| 17.5 | 24.73 | 24.85 | 29.70 | 29.84 |
| 18 | 25 | 25 | 30 | 30 |

BMI: body mass index.

Supplementary Table 2 Sensitivity analysis of interpolation data

| Characteristics | Missing data (%) | After interpolation | Before interpolation | Statistics | *P* |
| --- | --- | --- | --- | --- | --- |
| Age, years | 0.0 |  |  |  |  |
| Gender | 0.0 |  |  |  |  |
| Height | 0.0 |  |  |  |  |
| Weight | 0.0 |  |  |  |  |
| Geographic region* | 0.0 |  |  |  |  |
| Residential areas | 0.0 |  |  |  |  |
| Maternal education level | 0.0 |  |  |  |  |
| Smoking | 0.0 |  |  |  |  |
| Alcohol consumption | 0.0 |  |  |  |  |
| Hipline, Mean ± SD | 1.2 | 84.65 ± 9.82 | 84.64 ± 9.83 | t=0.04 | 0.966 |
| Waistline, Mean ± SD | 1.0 | 69.63 ± 9.96 | 69.59 ± 9.91 | t=0.17 | 0.862 |
| SBP, Mean ± SD | 4.8 | 106.05 ± 11.49 | 106.06 ± 11.52 | t=-0.02 | 0.981 |
| DBP, Mean ± SD | 4.8 | 69.53 ± 8.52 | 69.57 ± 8.55 | t=-0.15 | 0.878 |
| Diet knowledge | 0.0 |  |  |  |  |

SD: standard deviation; SBP, systolic blood pressure; DBP, diastolic blood pressure.

* Eastern China includes: the city of Beijing and Shanghai, the province of Jiangsu, Shandong and Zhejiang; Central China includes: the province of Henan, Hubei and Hunan; Western China includes: the province of Guizhou, Guangxi, Shanxi and Yunnan, the city of Chongqing; Northeastern China includes: the province of Liaoning and Heilongjiang.

Supplementary Table 3 The association between diet knowledge and overweight and obesity in children and adolescents

|  | Model 1 | |  | Model 2 | |  | Model 3 | |
| --- | --- | --- | --- | --- | --- | --- | --- | --- |
|  | OR (95%CI) | *P* |  | OR (95%CI) | *P* |  | OR (95%CI) | *P* |
| Overweight |  |  |  |  |  |  |  |  |
| Cluster B | Ref |  |  | Ref |  |  | Ref |  |
| Cluster A | 0.59 (0.42-0.83) | 0.002 |  | 0.59 (0.42-0.83) | 0.002 |  | 0.61 (0.43-0.88) | 0.007 |
| Cluster C | 1.13 (0.82-1.56) | 0.444 |  | 1.07 (0.78-1.48) | 0.673 |  | 1.14 (0.81-1.61) | 0.451 |
| Obesity |  |  |  |  |  |  |  |  |
| Cluster B | Ref |  |  | Ref |  |  | Ref |  |
| Cluster A | 0.28 (0.11-0.72) | 0.008 |  | 0.28 (0.11-0.73) | 0.009 |  | 0.29 (0.11-0.78) | 0.015 |
| Cluster C | 2.01 (1.14-3.54) | 0.015 |  | 1.90 (1.08-3.35) | 0.027 |  | 2.16 (1.18-3.93) | 0.012 |

Ref, reference; OR: odds ratio; CI: confidence interval; Cluster A: high diet knowledge level; Cluster B: medium diet knowledge level; Cluster C: low diet knowledge level.

Model 1: Single factor logistic regression analysis;

Model 2: Adjustment for age and gender;

Model 3: Adjustment for age, gender, geographic region, maternal education level, alcohol consumption, waist-to-hip ratio, systolic blood pressure and diastolic blood pressure

**References**

1. Cole TJ. Establishing a standard definition for child overweight and obesity worldwide: international survey. BMJ. 2000.
